# Enhanced Angle-of-Arrival and Polarization Parameter Estimation Using Localized Hybrid Dual-Polarized Arrays

**DOI:** 10.3390/s22145207

**Published:** 2022-07-12

**Authors:** Xiaolu Yu, Hang Li, Jian Andrew Zhang, Xiaojing Huang, Zhiqun Cheng

**Affiliations:** 1 School of Electronics and Information, Hangzhou Dianzi University, Hangzhou 310018, China; xiaoluyu@hdu.edu.cn (X.Y.); Zhiqun@hdu.edu.cn (Z.C.); 2 The Global Big Data Technologies Centre, University of Technology Sydney, Sydney 2007, Australia; andrew.zhang@uts.edu.au (J.A.Z.); xiaojing.huang@uts.edu.au (X.H.)

**Keywords:** hybrid dual-polarized array, localized subarrays, angle-of-arrival estimation, polarization parameter, mmWave communications

## Abstract

The millimeter wave (mmWave) channel is dominated by line-of-sight propagation. Therefore, the acquisition of angle-of-arrival (AoA) and polarization state of the wave is of great significance to the receiver. In this paper, we investigate AoA and polarization estimation in a mmWave system employing dual-polarized antenna arrays. We propose an enhanced AoA estimation method using a localized hybrid dual-polarized array for a polarized mmWave signal. The use of dual-polarized arrays greatly improves the calibration of differential signals and the signal-to-noise ratio (SNR) of the phase offset estimation between adjacent subarrays. Given the estimated phase offset, an initial AoA estimate can be obtained, and is then used to update the phase offset estimation. This leads to a recursive estimation with improved accuracy. We further propose an enhanced polarization estimation method, which uses the power of total received signals at dual-polarized antennas to compute the cross-correlation-to-power ratio instead of using only one axis dipole. Thus the accuracy of polarization parameter estimation is improved. We also derive a closed-form expression for mean square error lower bounds of AoA estimation and present an average SNR analysis for polarization estimation performance. Simulation results demonstrate the superiority of the enhanced AoA and polarization parameter estimation methods compared to the state of the art.

## 1. Introduction

With its large available bandwidth, millimeter wave (mmWave) system is a promising candidate for future cellular communications. It also plays a crucial role in other applications such as high-rate connections in vehicular networks [1,2,3,4,5]. In contrast to microwave channels that are characterized by extensive scattering, the mmWave channels have limited scattering and diminished diffraction. This enables line-of-sight (LOS) propagation typically to take dominance compared to non-LOS (NLOS) conditions when the wave arrives at its destination. Therefore, the acquisition of AoA and polarization state of the wave is critical for signal reception. On the other hand, as a result of the polarization mismatch between the receiving antennas and the incoming polarized wave, the use of single-polarized hybrid arrays often leads to a loss of signal power and thus poor AoA estimation accuracy. Therefore, hybrid dual-polarized array [6,7,8] is considered as an effective method for improving the AoA estimation performance and immunizing the signal-to-noise (SNR) degradation.

Hybrid antenna array is a new type of array structure for future mmWave high-speed communications, and it has the advantage that the phase shifting values and digital weights can be flexibly changed to jointly optimize the performance [4]. Moreover, compared with full digital array, hybrid antenna array is more applicable to energy saving systems due to its low power consumption [9,10]. Typically, a hybrid antenna array consists of multiple analog subarrays with phase-tunable antenna elements. It is classified into two types of regular configurations, i.e., localized and interleaved arrays in terms of antenna distribution in a subarray [11,12]. In view of the complexity of analog hardware implementation, the localized array can easily form a larger array by combining multiple subarrays into assembly modules, and thus is more applicable in practice.

AoA estimation using a localized array has been widely studied in [12,13,14,15,16,17]. All of them exploit the cross correlations between consecutive subarrays to extract the AoA information, where the phase ambiguity problem remains to be solved. The phase ambiguity results from an unknown integer multiple of 2π difference between *N* times of an AoA information (i.e., Nu) and the argument of the cross correlations, where *N* is the number of antennas in a subarray and *u* denotes the AoA information parameter. The works in [12,13,14] leveraged the same phase shift configuration across different subarrays for constructive accumulation of cross correlations. In [13], a differential beam searching algorithm was proposed to search all possible beams and select the estimate with the maximum output power by iterations, while it introduces a long estimation delay and suffers from a zigzag effect. An adaptive searching and tracking algorithm was then developed in [12] to speed up the searching process and mitigate the zigzag effect. To avoid this delay, a frequency-domain AoA estimation algorithm applied to a wideband array was proposed in [14], whereas the noise induced by the conjugate product of cross correlations is badly amplified.

In [15], the authors proposed a subarray based variable phase shift configuration that allows AoA estimation without the phase ambiguity. The proposed approach estimates Nu by calibrating the signs of cross correlations and combining them in a constructive way. By suppressing $e^{jmNu}$ in the *m*th subarray output signal, one can take its inverse discrete Fourier transform (IDFT), and then calculate the correlations of the Fourier coefficients to unambiguously estimate *u*. Furthermore, the authors in [16,17] extended the phase shift designs of [15] to narrowband and wideband systems respectively, and revealed that all cross correlations have the same signs except the strongest one. This discovery improves the calibration accuracy of cross correlations and thus coherent combining for estimating Nu. Without consideration of the polarization, all of the above works only study the AoA estimation using single-polarized hybrid arrays. However, the reception of a polarized wave has a practically significant impact on AoA estimation accuracy due to polarization mismatch.

The knowledge of the polarization state is important for assisting beam alignment and combining polarization diversity at the receiver. The polarization parameter estimation using dual-polarized arrays has received extensive attention recently, and several classical estimation methods have been proposed and studied in depth. In [18], the authors used rotational invariance techniques (ESPRIT) to estimate the polarization parameters of the incoming wave for a uniform linear array, and the methods were then extended to a two-dimensional array in [19]. A uni-vector-sensor ESPRIT based method was proposed in [20] to estimate polarization parameters and AoA using one electromagnetic vector sensor, which measures six electromagnetic field components of the incident wave field. Although this method has no frequency-AOA ambiguity and eliminates array interelement calibration, it only allows the use of one independent vector sensor. The ESPRIT method can achieve high parameter estimation accuracy, but the computational complexity introduced by the covariance matrix and singular value decomposition is proportional to the cube of the number of antennas. Therefore, it is only suitable for a small number of antenna arrays [18,19,20]. We proposed cross-correlation-to-power ratio polarization tracking algorithms in [8] that only require multiplication and addition operations on scalars. Although they have lower computational complexity than those based on ESPRIT, using only *x*- or *y*-axis dipoles for cross-correlation-to-power ratio calculation causes a loss in estimation performance. A parallel co-prime polarization sensitive array (PCP-PSA) was proposed to estimate two-dimensional AoA and polarization parameters in [21]. Compared to the PSA algorithm, it improves the accuracy of polarization parameter estimation by increasing the degrees of freedoms (DOFs). Furthermore, a three-parallel co-prime polarization sensitive array (TPCP-PSA) in [22] was used to obtain more DOFs for estimation improvement.

In this paper, we study the reception of a polarized mmWave signal using localized hybrid dual-polarized arrays, and propose novel algorithms for estimating AoA and polarization parameters. Simulation results show that they can achieve significantly improved performance compared to existing schemes. The main contributions of this paper are summarized below.

1. We develop an enhanced AoA estimation algorithm for dual-polarized arrays, which effectively improves the calibration of differential signals and thus the estimate of phase offset between adjacent subarrays. Given the phase offset, an initial AoA estimate can be obtained and used to update the phase offset estimation, which improves the accuracy of AoA estimation.
2. To improve the accuracy of the polarization parameter estimation, based on the AoA estimation results, we further propose a cross-correlation-to-power ratio based estimation approach, which exploits the total received power of signals for ratio calculation instead of only using one axis dipole. Furthermore, the proposed approach calculates the cross-correlation and power before digital beamforming rather than after digital beamforming in [8], which can achieve a higher average SNR for polarization estimation.
3. To evaluate the estimation performance, we derive the mean square error lower bounds (MSELBs) of AoA estimation, and analyse the average SNRs for polarization parameter estimation. It is analytically shown that the proposed approach has higher average SNRs than that in [8].

The rest of this paper is organized as follows. Section 1 introduces the received signal models for a hybrid dual-polarized array. Section 2 and Section 3 present the enhanced AoA estimation and polarization estimation approaches for a linear array, respectively, and then for a uniform planar array. Section 4 derives the MSELBs of AoA estimation and analyses the average SNRs for polarization parameter estimation. In Section 5, numerical and simulation results are given to demonstrate the performance of the proposed estimation approaches, before concluding the paper in Section 6.

**Notions:** mod{·,·} stands for the modulo operation. ${\left(\cdot \right)}^{T}$, ${\left(\cdot \right)}^{*}$ and |(·)| represent the transpose, conjugate and absolute value of (·), respectively. arg{·} denotes the argument of the complex number (·). sign{(·)} denotes taking the sign of (·).

## 2. System Models

### Hybrid Dual-Polarized Array with Localized Subarrays

**Figure 1:**
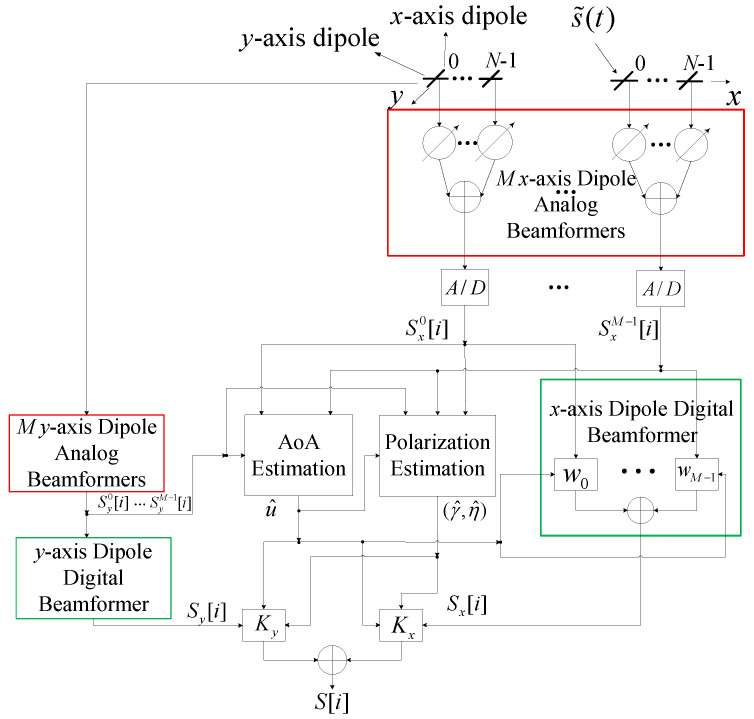
Illustration of linear localized hybrid dual-polarized antenna arrays.

As shown in Figure 1, we consider a uniform linear hybrid dual-polarized array with *M* subarrays, each including *N* electromagnetic vector sensors (EMVS) [1,2,3,4]. Each EMVS has two orthogonal dipoles collocated along *x*- and *y*-directions, called *x*- and *y*-axis dipoles respectively. They are used to measure the components of the incoming electric field projected onto the directions of *x*- and *y*-axes. The signals from the *x*- and *y*-axis dipoles of each subarray are combined after phase shifter to form the subarray output signals, respectively, followed by analog-to-digital (A/D) conversion. The digital signals from the *x*- and *y*-axes are jointly used to estimate the AoA information and polarization parameters, and are weighted and summed to form the output signals, respectively. In Figure 1, the radio frequency and down conversion modules are suppressed for simplicity. Note that the module named “*M* *y*-axis Dipole Analog Beamformers” shares the same signal processing with the one named “*M* *x*-axis Dipole Analog Beamformers” shown in the red box. Likewise, the module named “*y*-axis Dipole Digital Beamforemer” has the same components with “*x*-axis Dipole Digital Beamforemer” in the green box.

Suppose the reception of a transverse electromagnetic wave signal $\tilde{s}\left(t\right)$ with an elevation angle of θ and a polarization state of (γ,η) generated by its electric field [23]. $\gamma \in [0,\frac{\pi }{2})$ and η∈[−π,π) represent the auxiliary polarization angle and the polarization phase difference, respectively, which uniquely determine the polarization state of the wave. For example, η=0 refers to linearly-polarized waves, while $\gamma =\frac{\pi }{4}$ and $\eta =\pm \frac{\pi }{2}$ refer to left/right circularly-polarized wave. Therefore, the electric field vector e is expressed in Cartesian coordinates as: $e=e_{x}v_{x}+e_{y}v_{y}+e_{z}v_{z}$, where v is a unit vector along the subscript’s coordinate, and $\left[e_{x},e_{y},e_{z}\right]=\left[\sin\gamma \cos\theta e^{j\eta },\cos\gamma ,-\sin\gamma \sin\theta e^{j\eta }\right]$ denote the responses of the corresponding subscripts. After down-conversion, the received signal through the *m*th subarray (m=0,1,…,M−1) can be written as

(1) $$\begin{array}{cc} = & \left[e_{x},e_{y}\right]\tilde{s}\left(t\right)P_{t}^{m}\left(u\right)e^{jmNu}+\left[n_{x}^{m}\left(t\right),n_{y}^{m}\left(t\right)\right], \end{array}$$

where $s_{x}^{m}\left(t\right)$ and $s_{y}^{m}\left(t\right)$ are the signals received by the *x*- and *y*-axis dipoles, respectively. In (1), $P_{t}^{m}\left(u\right)$ denotes the radiation pattern of the *m*th subarray at time *t*, and is expressed as

(2) $$\begin{array}{c} P_{t}^{m}\left(u\right)=\sum_{n=0}^{N-1}{\overset{ˇ}{P}}_{t}^{m}\left(u\right)e^{j(nu+\alpha_{t}^{m}\left(n\right))}, \end{array}$$

where ${\overset{ˇ}{P}}_{t}^{m}\left(u\right)$ denotes the radiation pattern of the *n*th EMVS (n=0,1,…,N−1) at the *m*th subarray, and we assume ${\overset{ˇ}{P}}_{n}^{m}\left(u\right)=1$ in this paper; $\alpha_{t}^{m}\left(n\right)$ is the phase shift at the *n*th EMVS and $u=\frac{2\pi }{\lambda }d\sin\theta$; λ is the wavelength of the carrier, and *d* denotes the spacing between two adjacent EMVS; and $n_{x}^{m}\left(t\right)$ and $n_{y}^{m}\left(t\right)$ are the zero-mean additive white Gaussian noise (AWGN) along *x*- and *y*-axis dipoles at the *m*th subarray with the same power $\sigma_{n}^{2}$.

The outputs of analog beamformers, $[s_{x}^{m}\left(t\right)$,$s_{y}^{m}\left(t\right)]$, are then converted into digital signals, $[s_{x}^{m}\left[i\right]$,$s_{y}^{m}\left[i\right]]$, via A/D converter, where t=iT and *T* represents the sampling interval equalling the width of a symbol. As a result, the outputs of digital beamformers from *x*- and *y*-axis dipoles can be obtained by weighted summation as

(3) $$\begin{array}{c} \left[s_{x}\left[i\right],s_{y}\left[i\right]\right]=\sum_{m=0}^{M-1}w_{m}\left[s_{x}^{m}\left[i\right],s_{y}^{m}\left[i\right]\right], \end{array}$$

where $w_{m}$ is the digital weight at the *m*th subarray for aligning the phase of signals; $\left[s_{x}\left[i\right],s_{y}\left[i\right]\right]$ are used to produce s[i] with the maximum signal power by exploiting the MRC; and s[i] can be written as

(4) $$s\left[i\right]=k_{x}s_{x}\left[i\right]+k_{y}s_{y}\left[i\right],$$

with $\left[k_{x},k_{y}\right]$ denoting the MRC coefficients.

## 3. Enhanced Aoa Estimation Approach

In this section, we apply the phase shift designs in [16] to hybrid dual-polarized arrays, and propose an enhanced AoA estimation approach for improving the accuracy of phase offset estimation between adjacent subarrays. The initial AoA estimate can be acquired through the estimated phase offset, and is then used for the update of phase offset estimation. This improves the accuracy of AoA estimation recursively.

### 3.1. Estimation of Nu

As in [16], the *n*th (n=0,1,…,N−1) phase shift of the *m*th (m=0,1,…,M−1) subarray at the *i*th (i=0,1,…,I−1) symbol can be expressed as

(5) $$\begin{array}{c} \alpha_{i}^{m}\left(n\right)=-n\alpha_{i}^{m}=\frac{-2\pi n(\;\mathrm{mod}\;\{m,K\}I+i)}{L}, \end{array}$$

where $\alpha_{i}^{m}$ represents the phase shift difference between any two adjacent EMVS of the *m*th subarray at the *i*th symbol, indicating that each subarray directs at a predefined direction; and *K* is the number of different phase shifts for any symbol. Let K∈(2,M] and N=QK, where *Q* is an integer; L=IK is the overall number of different phase shifts adopted in the system, where *I* is the number of reference signals and *L* is the total numbers of different phase shifts. In terms of the configuration given by (5), AoA acquisition can be guaranteed by using at least one of the *L* beams with high gain, where the *L* beams sweep *L* evenly distributed directions within [−π,π). mod{m,K} implies that the cycle of $\alpha_{i}^{m}$ occurs every *K* subarrays in a symbol.

By substituting (5) to (2) after the A/D converter, we have

(6) $$\begin{array}{cc} P_{i}^{m}\left(u\right) & =\sum_{n=0}^{N-1}e^{jn(u-\alpha_{i}^{m})}=e^{j\left(N-1\right)\omega_{i}^{m}}\frac{\sin(N\omega_{i}^{m})}{\sin(\omega_{i}^{m})}, \end{array}$$

where $\omega_{i}^{m}=\left(u-\alpha_{i}^{m}\right)/2$. When the first *I* subarrays are considered, $\omega_{i}^{m}$ can be simplified as $\omega_{i}^{m}=\frac{u}{2}-\pi \left(\frac{m}{K}+\frac{i}{L}\right)$.

Computing the differential signals between the output signals of the *m*th and (m+1)th subarrays at *i*th symbol along *x*- and *y*-axis generates

(7) $$\begin{array}{cc} {}[\rho_{x}^{m}\left[i\right],\rho_{y}^{m}\left[i\right]]= & \left[{\left(s_{x}^{m}\left[i\right]\right)}^{*}s_{x}^{m+1}\left[i\right],{\left(s_{y}^{m}\left[i\right]\right)}^{*}s_{y}^{m+1}\left[i\right]\right]\\ = & \underset{\mathrm{signal}\mathrm{component}}{\underset{︸}{\left[\right|e_{x}{|}^{2},|e_{y}{|}^{2}]\cdot |\tilde{s}{\left[i\right]|}^{2}G_{i}^{m}\left(u\right)e^{jNu}}}+\left[z_{x}^{m}\left[i\right],z_{y}^{m}\left[i\right]\right], \end{array}$$

where

(8) $$\begin{array}{cc} G_{i}^{m}\left(u\right)= & {\left(P_{i}^{m}\left(u\right)\right)}^{*}P_{i}^{m+1}\left(u\right)\\ = & e^{\frac{-j(N-1)\pi }{K}}\frac{\sin\left(N\omega_{i}^{m}\right)\sin\left(N\omega_{i}^{m+1}\right)}{\sin\left(\omega_{i}^{m}\right)\sin\left(\omega_{i}^{m+1}\right)}\\ = & e^{\frac{-j(N-1)\pi }{K}}\frac{{\left(-1\right)}^{Q}\sin^{2}\left(N\omega_{i}^{m}\right)}{\sin\left(\omega_{i}^{m}\right)\sin\left(\omega_{i}^{m+1}\right)}. \end{array}$$

$\left[z_{x}^{m}\left[i\right],z_{y}^{m}\left[i\right]\right]$ are approximated as the zero-mean complex Gaussian noises given by

$$\begin{array}{cc} z_{l}^{m}\left[i\right]= & {\left(n_{l}^{m}\left[i\right]\right)}^{*}n_{l}^{m\;+\;1}\left[i\right]\;+\;e_{l}^{*}{\tilde{s}}^{*}\left[i\right]\left(P_{i}^{m}\left(u\right)\right){\;}^{*}\;e^{-jmNu}n_{l}^{m\;+\;1}\left[i\right]\\ \; & +e_{l}\tilde{s}\left[i\right]P_{i}^{m+1}\left(u\right)e^{j(m+1)Nu}{\left(n_{l}^{m}\left[i\right]\right)}^{*}.\;l\in \left\{x,y\right\} \end{array}$$

Note that $\rho_{x}^{m}\left[i\right]$ and $\rho_{y}^{m}\left[i\right]$ in (7) have an identical phase, Nu, in signal component, and their coherent summation has individual components added in phase. Therefore, we take a constructive combination of $\rho_{x}^{m}\left[i\right]$ and $\rho_{y}^{m}\left[i\right]$ to improve the accuracy of the estimate of Nu, $\hat{Nu}$. The complex gains of $G_{i}^{m}\left(u\right)$∀m,i in (7) are dependent on *u* and their signs are unknown in advance. This requires their signs to be calibrated and consistent for improving the accuracy of $\hat{Nu}$ by constructive combination.

Theorem 1 in [16] stated that for a single-polarized hybrid array at a symbol *i*, only $G_{i}^{m^{\prime }}\left(u\right)\neq 0$, with the largest amplitude, has the opposite sign to all the rest of $G_{i}^{m}\left(u\right)$, where $m,m^{\prime }\in \left[0,K-1\right]$ and $m\neq m^{\prime }$. As a result, we propose to find $m^{\prime }$ which is the index of $\rho_{x}^{m}\left[i\right]+\rho_{y}^{m}\left[i\right]$ with the largest amplitude, i.e.,

(9) $$\begin{array}{c} m^{\prime }=\underset{m=0:K-1}{\mathrm{argmax}}\left\{\left|\rho_{x}^{m}\left[i\right]+\rho_{y}^{m}\left[i\right]\right|\right\}. \end{array}$$

Given $m^{\prime }$, the signs of the differential signals can be aligned following

(10) $$\begin{array}{c} \left[{\tilde{\rho }}_{x}^{m}\left[i\right],{\tilde{\rho }}_{y}^{m}\left[i\right]\right]=\left\{\begin{array}{cc} & {\left(-1\right)}^{Q}\left[\rho_{x}^{m}\left[i\right],\rho_{y}^{m}\left[i\right]\right],\;\;\;\;\;\;\;\;\;\;\;\;m\neq m^{\prime }\\ & {\left(-1\right)}^{Q+1}\left[\rho_{x}^{m^{\prime }}\left[i\right],\rho_{y}^{m^{\prime }}\left[i\right]\right],\;\;\;m=m^{\prime } \end{array}\right. \end{array}$$

to perform in-phase combination for $\hat{Nu}$. In addition, we combine ${\tilde{\rho }}_{x}^{m}\left[i\right]$ and ${\tilde{\rho }}_{y}^{m}\left[i\right]$ across all subarrays and symbols constructively to improve the accuracy of $\hat{Nu}$ as shown in Algorithm 1, where l∈{x,y}.

Note that Algorithm 1 in [16] is basically similar to Steps 1–10 of our Algorithm 1. The difference is that the acquisition of $m^{\prime }$ in Step 6 and $\hat{Nu}$ in Step 10 exploits the coherent combination of the signals from two axes, instead of one axis in Algorithm 1 of [16]. Therefore, our algorithm can improve the performance of identifying the correct $m^{\prime }$ by coherently merging the differential signals of EMVS, thus effectively suppressing the noise and indirectly improving the estimation SNR. This will be validated in simulation results. At Step 10, the aligned differential signals from dual dipoles are also coherently combined to directly improve the estimation SNR compared to that in [16].

### 3.2. Estimation of *u*

Given $\hat{Nu}$, one can calibrate the output signals of subarrays, $\left[s_{x}^{m}\left[i\right],s_{y}^{m}\left[i\right]\right]$, by multiplying $e^{-jm\hat{Nu}}$, i.e., $\left[s_{x}^{m}\left[i\right],s_{y}^{m}\left[i\right]\right]e^{-jm\hat{Nu}}$. Assuming that $e^{jm(Nu-\hat{Nu})}\approx 1$, $\left[s_{x}^{m}\left[i\right],s_{y}^{m}\left[i\right]\right]$ can be almost perfectly calibrated. Then taking the *K*-point IDFTs of $\left[s_{x}^{m}\left[i\right],s_{y}^{m}\left[i\right]\right]e^{-jm\hat{Nu}}$ from the adjacent *K* subarray outputs, we can obtain $\left[{\tilde{S}}_{x}^{k}\left[i\right],{\tilde{S}}_{y}^{k}\left[i\right]\right]$, m,k=0,…,K−1, given by

(11) $$\begin{array}{c} \left[{\tilde{S}}_{x}^{k}\left[i\right],{\tilde{S}}_{y}^{k}\left[i\right]\right]\approx \left[e_{x},e_{y}\right]\cdot \tilde{s}\left[i\right]p_{i}^{k}\left(u\right)+\left[N_{x}^{k}\left[i\right],N_{y}^{k}\left[i\right]\right], \end{array}$$

where

$$\begin{array}{c} p_{i}^{k}\left(u\right)=e^{j[k\left(u-\frac{2\pi i}{L}\right)+\left(N-K\right)\left(\frac{u}{2}-\frac{\pi i}{L}\right)]}\frac{\sin\left(\frac{Nu}{2}-\frac{N\pi i}{L}\right)}{\sin\left(\frac{Ku}{2}-\frac{K\pi i}{L}\right)} \end{array}$$

are the Fourier coefficients of $P_{i}^{m}\left(u\right)$ and $\left[N_{x}^{k}\left[i\right],N_{y}^{k}\left[i\right]\right]$ are the *K*-point IDFTs of $\left[n_{x}^{m}\left[i\right],n_{y}^{m}\left[i\right]\right]e^{-jm\hat{Nu}}$.
**Algorithm 1** Enhanced AoA Estimation**Input:** $\left[s_{x}^{m}\left[i\right],s_{y}^{m}\left[i\right]\right]$, m=0:M−1, i=0:I−1;**Output:** $\hat{u}$; 1:**for**i=0:I−1**do** 2:    Calculate $\left[\rho_{x}^{m}\left[i\right],\rho_{y}^{m}\left[i\right]\right]$ by (7), m=0:M−2; 3:    **if** K=M **then** 4:        $\rho_{l}^{K-1}\left[i\right]\leftarrow {\left(s_{l}^{K-1}\left[i\right]\right)}^{*}s_{l}^{0}\left[i\right]$; 5:    **end if** 6:    Determine $m^{\prime }$ by (9); 7:    ${\tilde{\rho }}_{l}^{m}\left[i\right]\leftarrow {\left(-1\right)}^{Q}\rho_{l}^{m}\left[i\right]$, m=0:M−2; 8:    ${\tilde{\rho }}_{l}^{m}\left[i\right]\leftarrow -{\tilde{\rho }}_{l}^{m}\left[i\right]$, $m=m^{\prime }:K:M-2$; 9:**end for**10:$\hat{Nu}\leftarrow \arg\left\{e^{\frac{j(N-1)\pi }{K}}\sum_{i=0}^{I-1}\sum_{m=0}^{M-2}\left({\tilde{\rho }}_{x}^{m}\left[i\right]+{\tilde{\rho }}_{y}^{m}\left[i\right]\right)\right\}$;11:**for**i=0:I−1**do**12:    ${\tilde{s}}_{l}^{m}\left[i\right]\leftarrow s_{l}^{m}\left[i\right]e^{-jm\hat{Nu}}$, m=0:M−1;13:    **for** q=0:⌊M/K⌋−1 **do**14:        ${\tilde{s}}_{l}^{q}\left[i\right]\leftarrow \left\{{\tilde{s}}_{l}^{qK}\left[i\right],{\tilde{s}}_{l}^{qK+1}\left[i\right],\ldots ,{\tilde{s}}_{l}^{(q+1)K-1}\left[i\right]\right\}$;15:        ${\tilde{S}}_{l}^{q}\left[i\right]\leftarrow \mathrm{IDFT}\left\{{\tilde{s}}_{l}^{q}\left[i\right]\right\}$;16:    **end for**17:    ${\tilde{s}}_{l}^{\left\lfloor M/K\right\rfloor }\left[i\right]\leftarrow \left\{{\tilde{s}}_{l}^{\lfloor M/K\rfloor K}\left[i\right],\ldots ,{\tilde{s}}_{l}^{M-1}\left[i\right],\right.$18:                         $\left.{\tilde{s}}_{l}^{M-\lfloor M/K\rfloor K}\left[i\right],\ldots ,{\tilde{s}}_{l}^{K-1}\left[i\right]\right\}$;19:    ${\tilde{S}}_{l}^{\left\lfloor M/K\right\rfloor }\left[i\right]\leftarrow \mathrm{IDFT}\left\{{\tilde{s}}_{l}^{\left\lfloor M/K\right\rfloor }\left[i\right]\right\}$;20:**end for**21:$H\left[i\right]\leftarrow \sum_{q=0}^{\left\lfloor M/K\right\rfloor }\sum_{l\in \{x,y\}}{\left({\tilde{S}}_{l}^{q}\left[i\right]\right)}_{\left(1:K-1\right)}^{*}{\left({\tilde{S}}_{l}^{q}\left[i\right]\right)}_{\left(2:K\right)}^{T}$;22:$\hat{u}\leftarrow \arg\left\{\sum_{i=0}^{I-1}e^{\frac{j2\pi i}{L}}H\left[i\right]\right\}$;23:$\hat{Nu}\leftarrow N\hat{u}$, and **repeat** Steps 11–22.

To obtain an unambiguous estimation of *u*, we compute the cross-correlation between any two adjacent IDFT outputs, $\left[{\left({\tilde{S}}_{x}^{k}\left[i\right]\right)}^{*}{\tilde{S}}_{x}^{k+1}\left[i\right],{\left({\tilde{S}}_{y}^{k}\left[i\right]\right)}^{*}{\tilde{S}}_{y}^{k+1}\left[i\right]\right]$. The differential signals, denoted by $\left[d_{x}^{k}\left[i\right],d_{y}^{k}\left[i\right]\right]$, k=0,…,K−2, can be written as

(12) $$\begin{array}{c} \left[d_{x}^{k}\left[i\right],d_{y}^{k}\left[i\right]\right]\;=\;\underset{\mathrm{signal}\mathrm{components}}{\underset{︸}{\left[\right|e_{x}{|}^{2}\;,\;|e_{y}{|}^{2}]|\tilde{s}{\left[i\right]|}^{2}\;{\left|\;\frac{\sin\left(\frac{Nu}{2}\;-\;\frac{N\pi i}{L}\right)}{\sin\left(\frac{Ku}{2}\;-\;\frac{K\pi i}{L}\right)}\;\right|}^{2}\;e^{j(u\;-\;\frac{2\pi i}{L})}}}+\left[{\tilde{N}}_{x}^{k}\left[i\right],{\tilde{N}}_{y}^{k}\left[i\right]\right], \end{array}$$

where

$$\begin{array}{c} {\tilde{N}}_{l}^{k}\left[i\right]={\left(N_{l}^{k}\left[i\right]\right)}^{*}N_{l}^{k+1}\left[i\right]+e_{l}^{*}{\tilde{s}}^{*}\left[i\right]{\left(p_{i}^{k}\left(u\right)\right)}^{*}N_{l}^{k+1}\left[i\right]+e_{l}\tilde{s}\left[i\right]p_{i}^{k+1}\left(u\right){\left(N_{l}^{k}\left[i\right]\right)}^{*} \end{array}$$

can be approximated as complex Gaussian noises with zero means, and noise powers $\sigma_{{\tilde{N}}_{l}}^{2}=2|e_{l}{|}^{2}|\tilde{s}{\left[i\right]|}^{2}{\left|p_{i}^{k}\left(u\right)\right|}^{2}\sigma_{N_{l}}^{2}$ when ignoring the higher-order term of $N_{l}^{k}\left[i\right]N_{l}^{k+1}\left[i\right]$. Here, $\sigma_{N_{l}}^{2}$ denotes noise power of $N_{l}^{k}\left[i\right]$(l∈{x,y}) given by $\sigma_{N_{l}}^{2}=\sigma_{n}^{2}/K$. It is seen from (12) that the estimate of *u*, $\hat{u}$, can be unambiguously obtained by $\hat{u}=\arg\left\{\left(d_{x}^{k}\left[i\right]+d_{y}^{k}\left[i\right]\right)e^{\frac{j2\pi i}{L}}\right\}$. Similarly, $\left[d_{x}^{k}\left[i\right],d_{y}^{k}\left[i\right]\right]$ over all subarrays and symbols can be constructively combined to improve the accuracy of $\hat{u}$.

Note that Algorithm 2 in [16] is basically similar to Steps 11–22 in our Algorithm 1. The difference is that in Step 23, we further propose to use $\hat{u}$ to update $\hat{Nu}$, i.e., $\hat{Nu}\leftarrow N\hat{u}$, and in turn to re-estimate $\hat{u}$. As a result, the estimation accuracy of *u* is improved after one iteration due to the upgraded $\hat{Nu}$. The improved MSE performance of $\hat{Nu}$ and the resulting $\hat{u}$ will be shown in the simulation results. The enhanced AoA estimation algorithm is summarized in Algorithm 1, where ${\left({\tilde{S}}_{l}^{q}\left[i\right]\;\right)}_{\left(k_{1}:k_{2}\right)}$ denotes the vector consisting of the $k_{1}$th to $k_{2}$th elements of ${\tilde{S}}_{l}^{q}\left[i\right]$.

### 3.3. Extension to Uniform Planar Array

The proposed algorithm can be readily extended from uniform linear arrays to planar arrays. When ignoring the noise at the receiver, the received signals after A/D converter at the $\left(m_{x},m_{y}\right)$th analog subarray $\left(m_{x}=0,1,\ldots ,M_{x}-1;m_{y}=0,1,\ldots ,M_{y}-1\right)$, where $M_{x}$ and $M_{y}$ are the numbers of subarrays along *x*- and *y*-axis directions, can be expressed as

(13) $$\begin{array}{c} {}[s_{x}^{m_{x},m_{y}}\left[i\right],s_{y}^{m_{x},m_{y}}\left[i\right]]=\left[e_{x},e_{y}\right]\tilde{s}\left[i\right]P_{i}^{m_{x},m_{y}}\left(u_{x},u_{y}\right)\times e^{j(m_{x}N_{x}u_{x}+m_{y}N_{y}u_{y})}, \end{array}$$

where $u_{x}=\frac{2\pi }{\lambda }d\sin\theta \cos\phi$, $u_{y}=\frac{2\pi }{\lambda }d\sin\theta \sin\phi$, and ϕ represents the azimuth angle of the received signal. The estimates of (θ,ϕ) is equivalent to those of $\left(u_{x},u_{y}\right)$, i.e., $\theta =\mathrm{sign}\left\{u_{x}\right\}\sin^{-1}\left(\frac{\lambda }{2\pi }\frac{\sqrt{u_{x}^{2}+u_{y}^{2}}}{Nd}\right)$ and $\phi =\tan^{-1}\left(\frac{u_{y}}{u_{x}}\right)$.

Let $N_{x}$ and $N_{y}$ be the numbers of EMVS in a subarray along the two directions. Extended from (5), the $\left(n_{x},n_{y}\right)$th $\left(n_{x}=0,1,\ldots ,N_{x}-1,n_{y}=0,1,\ldots ,N_{y}-1\right)$ phase shift of the $\left(m_{x},m_{y}\right)$th subarray at the *i*th symbol is given by

(14) $$\begin{array}{cc} \alpha_{i}^{m_{x},m_{y}}\left(n_{x},n_{y}\right)= & -2\pi n_{x}\left(\;\mathrm{mod}\;\left\{m_{x},K_{x}\right\}/K_{x}+i/L_{x}\right)\\ \; & -2\pi n_{y}\left(\;\mathrm{mod}\;\left\{m_{y},K_{y}\right\}/K_{y}+i/L_{y}\right), \end{array}$$

where $\left(K_{x},K_{y}\right)$ and $\left(L_{x},L_{y}\right)$ are two-dimensional extensions of *K* and *L* along *x*- and *y*-axes, respectively.

The associated radiation pattern $P_{i}^{m_{x},m_{y}}\left(u_{x},u_{y}\right)$ is derived by

(15) $$\begin{array}{c} \begin{array}{c} P_{i}^{m_{x},m_{y}}\left(u_{x},u_{y}\right)=e^{j\left(N_{x}-1\right)w_{x,i}^{m_{x}}}\frac{\sin(N_{x}w_{x,i}^{m_{x}})}{\sin(w_{x,i}^{m_{x}})}\cdot e^{j\left(N_{y}-1\right)w_{y,i}^{m_{y}}}\frac{\sin(N_{y}w_{y,i}^{m_{y}})}{\sin(w_{y,i}^{m_{y}})}, \end{array} \end{array}$$

where $w_{x,i}^{m_{x}}=\frac{u_{x}}{2}-\pi \left(\frac{m_{x}}{M_{x}}+\frac{i}{L_{x}}\right)$ and $w_{y,i}^{m_{y}}=\frac{u_{y}}{2}-\pi \left(\frac{m_{y}}{M_{y}}+\frac{i}{L_{y}}\right)$ assuming $K_{x}=M_{x}$ and $K_{y}=M_{y}$.

Calculating the differential signals between the output signals of the $\left(m_{x},m_{y}\right)$th and $\left(m_{x}+1,m_{y}\right)$th subarray at the *i*th symbol, $\left[\rho_{x}^{m_{x}}\left[i\right],\rho_{x}^{m_{y}}\left[i\right]\right]$, we have the corresponding $G_{i}^{m_{x}}\left(u_{x},u_{y}\right)$ in (8), given by

(16) $$\begin{array}{cc} G_{i}^{m_{x}}\left(u_{x},u_{y}\right)\; & ={\left(P_{i}^{m_{x},m_{y}}\left(u_{x},u_{y}\right)\right)}^{*}P_{i}^{m_{x}+1,m_{y}}\left(u_{x},u_{y}\right)\\ \; & =e^{-j\frac{(N_{x}-1)\pi }{K_{x}}}\frac{{\left(-1\right)}^{Q_{x}}\sin^{2}\left(N_{x}w_{x,i}^{m_{x}}\right)}{\sin\left(w_{x,i}^{m_{x}}\right)\sin\left(w_{x,i}^{m_{x}+1}\right)}\cdot {\left|\frac{\sin(N_{y}w_{y,i}^{m_{y}})}{\sin(w_{y,i}^{m_{y}})}\right|}^{2}, \end{array}$$

where $Q_{x}$ is the extension of *Q* along the *x*-axis direction. It can be seen from (16) that since ${\left|\frac{\sin(N_{y}\omega_{y,i}^{m_{y}})}{\sin(\omega_{y,i}^{m_{y}})}\right|}^{2}>0$ assuming $\frac{\sin(N_{y}\omega_{y,i}^{m_{y}})}{\sin(\omega_{y,i}^{m_{y}})}\;\neq \;0$, the extension of (9) is still applicable here, i.e.,

(17) $$\begin{array}{c} m_{x}^{\prime }=\underset{m=0:K-1}{\mathrm{argmax}}\left\{\left|\sum_{m_{y}=0}^{M_{y}-1}\left(\rho_{x}^{m_{x}}\left[i\right]+\rho_{y}^{m_{x}}\left[i\right]\right)\right|\right\}. \end{array}$$

We constructively combine the outputs of all subarrays along *y*-axis directions to find $m_{x}^{{}^{\prime }}$ in order to improve the SNR of the differential signals. Given $m_{x}^{{}^{\prime }}$, the signs of $\left[\rho_{x}^{m_{x}}\left[i\right],\rho_{y}^{m_{x}}\left[i\right]\right]$ are aligned following (10) as $\left[{\tilde{\rho }}_{x}^{m}\left[i\right],{\tilde{\rho }}_{y}^{m}\left[i\right]\right]$. Therefore, $\hat{N_{x}u_{x}}$ can be obtained by

$$\begin{array}{c} \hat{N_{x}u_{x}}\;=\;\arg\left\{e^{\frac{j(N_{x}-1)\pi }{K_{x}}}\sum_{i=0}^{I-1}\sum_{m_{y}=0}^{M_{y}-1}\sum_{m_{x}=0}^{M_{x}-2}\left({\tilde{\rho }}_{x}^{m_{x}}\left[i\right]+{\tilde{\rho }}_{y}^{m_{x}}\left[i\right]\right)\right\}. \end{array}$$

Likewise, we can obtain $\hat{N_{y}u_{y}}$ by identifying $m_{y}^{{}^{\prime }}$. In a similar way to estimating *u* in Algorithm 1, we can unambiguously estimate $u_{x}$ and $u_{y}$ given $\hat{N_{x}u_{x}}$ and $\hat{N_{y}u_{y}}$.

## 4. Enhanced Polarization State Estimation Approach

In this section, we propose an enhanced polarization parameter estimation approach to improve the accuracy of polarization state estimation, where the cross-correlation-to-power ratio is employed to estimate polarization state, and the total received power of signals on *x*- and *y*-axes is used for the ratio calculation instead of that on only one axis in [8].

### 4.1. Estimation of Polarization State

Given $\hat{u}$, we allow the main beam of each subarray to be directed towards the estimated direction of incoming signals by adjusting subarray radiation pattern. This enables the maximum received signals power to improve the accuracy of polarization parameter estimation. Therefore, $\left[s_{x}^{m}\left[i\right],s_{y}^{m}\left[i\right]\right]$ in (1) can be rewritten as

(18) $$\begin{array}{c} \left[s_{x,m}^{\prime }\left[i\right],s_{y,m}^{\prime }\left[i\right]\right]=\left[e_{x},e_{y}\right]\tilde{s}\left[i\right]P_{s}\left(u\right)e^{jmNu}+\left[n_{x}^{m}\left[i\right],n_{y}^{m}\left[i\right]\right], \end{array}$$

where

(19) $$\begin{array}{c} P_{s}\left(u\right)=\sum_{n=0}^{N-1}e^{j(nu+\beta (n))}=\frac{\sin\frac{N(u-\hat{u})}{2}}{\sin\frac{u-\hat{u}}{2}} \end{array}$$

denotes the subarray radiation pattern when the phase shift in each subarray is chosen as $\beta \left(n\right)=-n\hat{u}$.

It is shown from (18) that the polarization state, (γ,η), is contained in $\left[e_{x},e_{y}\right]$, thus we propose to extract the polarization parameters by exploiting the relative values between the output signals from two axes dipoles. Firstly, computing the differential signals between $s_{x,m}^{\prime }\left[i\right]$ and $s_{y,m}^{\prime }\left[i\right]$, we have

(20) $$\begin{array}{c} q_{xy}^{m}\left[i\right]=s_{x,m}^{\prime }\left[i\right]{\left(s_{y,m}^{\prime }\left[i\right]\right)}^{*}=\underset{\mathrm{signal}\mathrm{component}}{\underset{︸}{e_{x}e_{y}^{*}|\tilde{s}{\left[i\right]|}^{2}{\left|P_{s}\left(u\right)\right|}^{2}}}+n_{xy}^{m}\left[i\right], \end{array}$$

where

$$\begin{array}{c} n_{xy}^{m}\left[i\right]=n_{x}^{m}\left[i\right]{\left(n_{y}^{m}\left[i\right]\right)}^{*}+n_{x}^{m}\left[i\right]e_{y}^{*}{\tilde{s}}^{*}\left[i\right]{\left(P_{s}\left(u\right)\right)}^{*}e^{-jmNu}+{\left(n_{y}^{m}\left[i\right]\right)}^{*}e_{x}\tilde{s}\left[i\right]P_{s}\left(u\right)e^{jmNu} \end{array}$$

is approximately zero-mean complex AWGN. The associated instaneous powers of $s_{x,m}^{\prime }\left[i\right]$ and $s_{y,m}^{\prime }\left[i\right]$ are given by

(21) $$\begin{array}{cc} {}[p_{x}^{m}\left[i\right],p_{y}^{m}\left[i\right]]= & \left[|s_{x,m}^{\prime }\left[i\right]{|}^{2},|s_{y,m}^{\prime }\left[i\right]{|}^{2}\right]\\ = & \underset{\mathrm{signal}\mathrm{component}}{\underset{︸}{\left[\right|e_{x}{|}^{2}\;,\;|e_{y}{|}^{2}]|\tilde{s}{\left[i\right]|}^{2}{\left|P_{s}\left(u\right)\right|}^{2}}}\;+\;\left[n_{xx}^{m}\left[i\right],n_{yy}^{m}\left[i\right]\right]+\left[\right|n_{x}^{m}{\left[i\right]|}^{2},|n_{y}^{m}\left[i\right]{|}^{2}], \end{array}$$

where $[\left[n_{xx}^{m}\left[i\right],n_{yy}^{m}\left[i\right]\right]$ are also the zero-mean complex AWGNs.

In the absence of noise component, the ratio of $q_{xy}^{m}\left[i\right]$ to $p_{x}^{m}\left[i\right]+p_{y}^{m}\left[i\right]$, denoted by U+jV, can be expressed as

(22) $$\begin{array}{c} U+jV=\frac{e_{x}e_{y}^{*}}{|e_{x}{|}^{2}+{\left|e_{y}\right|}^{2}}=\frac{\sin\gamma \cos\theta e^{j\eta }\cos\gamma }{\sin^{2}\gamma \cos^{2}\theta +\cos^{2}\gamma }=\frac{\tan\gamma \cos\theta e^{j\eta }}{\tan^{2}\gamma \cos^{2}\theta +1}, \end{array}$$

which leads to the polarization state given by

(23) $$\left\{\begin{array}{cc} \gamma = & \arctan\left\{\cos^{-1}\theta \cdot \frac{1\pm \sqrt{1-4(U^{2}+V^{2})}}{2\sqrt{U^{2}+V^{2}}}\right\}\\ \eta = & \arg\{U+jV\} \end{array}\right.,$$

where $\cos\theta =\sqrt{1-{\left(\frac{\lambda u}{2\pi d}\right)}^{2}}$.

In the presence of noise component, since $q_{xy}^{m}\left[i\right]$ and $p_{x}^{m}\left[i\right]+p_{y}^{m}\left[i\right]$ over all subarrays and symbols have in-phase signal component respectively, the ratio U+jV can be estimated by coherently combining $q_{xy}^{m}\left[i\right]$ and $p_{x}^{m}\left[i\right]+p_{y}^{m}\left[i\right]$ over them, i.e.,

(24) $$\begin{array}{c} U+jV=\frac{\sum_{i=0}^{I^{{}^{\prime }}-1}\sum_{m=0}^{M-1}q_{xy}^{m}\left[i\right]}{\sum_{i=0}^{I^{{}^{\prime }}-1}\sum_{m=0}^{M-1}\left(p_{x}^{m}\left[i\right]+p_{y}^{m}\left[i\right]\right)-2MI^{{}^{\prime }}\sigma_{n}^{2}}, \end{array}$$

to improve the SNR of estimation, where $2MI^{{}^{\prime }}\sigma_{n}^{2}$ is the expectation of $\sum_{i=0}^{I^{{}^{\prime }}-1}\sum_{m=0}^{M-1}\left(\right|n_{x}^{m}{\left[i\right]|}^{2}+|n_{y}^{m}\left[i\right]{|}^{2})$, and $I^{{}^{\prime }}$ is the number of reference signals used for combination. Unlike in [8] where the calculations of cross-correlation and power are performed after digital beamforming, we compute the ratio of cross-correlation to power before digital beamforming, which can lead to a higher SNR of polarization estimation, as will be illustrated in Section 5.2.

Note that from (24), we calculate the total power of signals on *x*- and *y*-axis dipoles in the denominator, instead of the power calculation on only one axis in [8], which can enhance the noise immunity and improve the estimation performance. However, it is seen from (23) that there are two possible solutions for γ, denoted by $\gamma_{c}$ (c=1,2). Therefore, an identification is needed to find the real estimate. As the maximum power of the combined signals from all subarrays’ outputs can be produced only when the real polarization state estimate is used to configure the MRC coefficients, we determine the real polarization estimate by comparing the MRC power. Given $\hat{u}$, $\hat{\gamma }$ and $\hat{\eta }$, we can obtain the MRC coefficients by

(25) $$\begin{array}{c} \left[\kappa_{x,c},\kappa_{y,c}\right]=\frac{\left[v_{x,c},v_{y,c}\right]}{\sqrt{|v_{x,c}{|}^{2}+{\left|v_{y,c}\right|}^{2}}}, \end{array}$$

where

$$\begin{array}{c} \left[v_{x,c},v_{y,c}\right]=\left[\sin\hat{\gamma_{c}}\cos\hat{\theta }e^{-j\hat{\eta }},\cos\hat{\gamma_{c}}\right]. \end{array}$$

s[i] in (4) can be rewritten as

(26) $$\begin{array}{c} s_{c}\left[i\right]=\sum_{i=0}^{I^{{}^{\prime }}-1}\sum_{m=0}^{M-1}w_{m}\left(\kappa_{x,c}s_{x,m}^{\prime }\left[i\right]+\kappa_{y,c}s_{y,m}^{\prime }\left[i\right]\right) \end{array}$$

where $w_{m}=\frac{1}{M}e^{-jm\hat{u}}$ for aligning the array. As a result, we determine the real $\hat{\gamma }$ using

(27) $$\begin{array}{c} \hat{\gamma }=\underset{\hat{\gamma_{c}},c=1,2}{\mathrm{argmax}}\left\{|s_{c}{\left[i\right]|}^{2}\right\}. \end{array}$$

The enhanced polarization parameter estimation algorithm is summarized in Algorithm 2.
**Algorithm 2** Enhanced Polarization Estimation**Input:** $\left[s_{x,m}^{\prime }\left[i\right],s_{y,m}^{\prime }\left[i\right]\right]$, m=0:M−1, $i=0:I^{{}^{\prime }}-1$;**Output:** $\hat{\gamma }$, $\hat{\eta }$; 1:**for**$i=0:I^{{}^{\prime }}-1$**do** 2:    Calculate $q_{x,y}^{m}\left[i\right]$ by (20) and $\left[p_{x}^{m}\left[i\right],p_{y}^{m}\left[i\right]\right]$ by (21),      m=0,…,M−1; 3:**end for** 4:Compute U+jV by (24); 5:Determine $\left(\hat{\gamma },\hat{\eta }\right)$ by (23), 6:Calculate $s_{c}\left[i\right]$ by (26); 7:Determine the real estimate $\hat{\gamma }$ by (27).

### 4.2. Extension to Planar Arrays

The proposed polarization state estimation approach can also be readily extended to a planar array. Assuming that $\left(\hat{\theta },\hat{\phi }\right)$ is obtained as shown in Section 3.3, we can rewrite (23) in two dimensions as

(28) $$\left\{\begin{array}{cc} \gamma = & \arctan\left\{A\cos^{-1}\hat{\theta }\right\}\\ \eta = & \mathrm{sign}\left\{V\right\}\arccos\left\{\frac{2U\;+\;\sin\left(2\hat{\phi }\right)}{2A\cos\left(2\hat{\phi }\right)}\;+\;\frac{A(2U\;-\;\sin\left(2\hat{\phi }\right))}{2\cos\left(2\hat{\phi }\right)}\right\} \end{array}\right.$$

where

$$A=\sqrt{\frac{1-4U^{2}+\left(1-4V^{2}\right)\cos^{2}\left(2\hat{\phi }\right)\pm 2\sqrt{1-4(U^{2}+V^{2})}\cos\left(2\hat{\phi }\right)}{{\left(2U-\sin\left(2\hat{\phi }\right)\right)}^{2}+{\left(2V\cos\left(2\hat{\phi }\right)\right)}^{2}}}.$$

$\cos\hat{\theta }$, $\cos(2\hat{\phi })$ and $\sin(2\hat{\phi })$ can be obtained from ${\hat{u}}_{x}$ and ${\hat{u}}_{y}$, respectively as

(29) $$\left\{\begin{array}{cc} \cos\hat{\theta }= & \sqrt{1-{\left(\frac{\lambda }{2\pi d}\right)}^{2}\left({\hat{u}}_{x}^{2}+{\hat{u}}_{y}^{2}\right)}\\ \cos(2\hat{\phi })= & \left({\hat{u}}_{x}^{2}-{\hat{u}}_{y}^{2}\right)/\left({\hat{u}}_{x}^{2}+{\hat{u}}_{y}^{2}\right)\\ \sin(2\hat{\phi })= & 2{\hat{u}}_{x}{\hat{u}}_{y}/\left({\hat{u}}_{x}^{2}+{\hat{u}}_{y}^{2}\right). \end{array}\right.$$

Similarly, the real polarization state can be determined by

(30) $$\begin{array}{c} \left(\hat{\gamma },\hat{\eta }\right)=\underset{(\hat{\gamma_{c}},\hat{\eta_{c}}),c=1,2}{\mathrm{argmax}}\left\{|s_{c}{\left[i\right]|}^{2}\right\}. \end{array}$$

### 4.3. Extension to EMVS with *z*-Axis Dipole

To collect extra power, an EMVS can be equipped with an additional *z*-axis dipole that is orthogonally collocated with the *x*/*y*-axes. $e_{z}=-\sin\gamma \sin\theta e^{j\eta }$ gives the associated response in an electric field. We use the received signal on the *z*-axis dipole to compute the differential signals between the *z*-axis and *x*/*y*-axes dipoles as

(31) $$\begin{array}{cc} {}[q_{yz}^{m}\left[i\right],q_{zx}^{m}\left[i\right]]= & \left[s_{y,m}^{\prime }\left[i\right]{\left(s_{z,m}^{\prime }\left[i\right]\right)}^{*},s_{z,m}^{\prime }\left[i\right]{\left(s_{x,m}^{\prime }\left[i\right]\right)}^{*}\right]\\ = & \left[e_{y}e_{z}^{*},e_{z}e_{x}^{*}\right]|\tilde{s}\left[i\right]{|}^{2}{\left|P_{s}\left(u\right)\right|}^{2}+\left[n_{yz}^{m}\left[i\right],n_{zx}^{m}\left[i\right]\right], \end{array}$$

and the instaneous power on *z*-axis dipole as

$$\begin{array}{c} p_{z}^{m}\left[i\right]=|s_{z,m}^{\prime }{\left[i\right]|}^{2}=|e_{z}{|}^{2}|\tilde{s}{\left[i\right]|}^{2}|P_{s}{\left(u\right)|}^{2}\;+\;n_{zz}^{m}\left[i\right]\;+\;{\left|n_{z}^{m}\left[i\right]\right|}^{2}. \end{array}$$

In noiseless circumstances, the ratio of the sum of differential signals to the sum of instaneous power can be derived as

$$\begin{array}{c} U+jV=\frac{e_{x}e_{y}^{*}+e_{y}e_{z}^{*}+e_{z}e_{x}^{*}}{|e_{x}{|}^{2}+|e_{y}{|}^{2}+{\left|e_{z}\right|}^{2}}=e_{x}e_{y}^{*}+e_{y}e_{z}^{*}+e_{z}e_{x}^{*}, \end{array}$$

which results in the polarization state represented by *U* and *V*. Similar to (24), U+jV can be estimated by

$$\begin{array}{c} \frac{\sum_{i=0}^{I^{{}^{\prime }}-1}\sum_{m=0}^{M-1}\left(q_{xy}^{m}\left[i\right]+q_{yz}^{m}\left[i\right]+q_{zx}^{m}\left[i\right]\right)}{\sum_{i=0}^{I^{{}^{\prime }}-1}\sum_{m=0}^{M-1}\left(p_{x}^{m}\left[i\right]+p_{y}^{m}\left[i\right]+p_{z}^{m}\left[i\right]\right)-3MI^{{}^{\prime }}\sigma_{n}^{2}}. \end{array}$$

Equation (27) is still applicable to determining the real one.

## 5. Estimation Performance Evaluation

In this section, we derive the closed-form MSELBs for the enhanced AoA estimation approach and present an analysis of the proposed polarization parameter estimation.

### 5.1. The MSELBs of AoA Estimation

We evaluate the AoA estimation performance of the proposed algorithm using the MSE of $\hat{u}$, and derive its closed-form MSELBs. If $\hat{Nu}=Nu$, i.e., $e^{jm(Nu-\hat{Nu})}=1$, all subarray output signals can be perfectly aligned. From (12) and Step 22 of Algorithm 1, the estimation of *u* is formulated as the phase estimation of the accumulation of $d_{x}^{qK+k}\left[i\right]+d_{y}^{qK+k}\left[i\right]$ over all *k*, *q* and *i* (denoted by *D*), given $\tilde{s}\left(t\right)$, *u*, γ and η (collectively denoted by c). For convenience of analysis, we consider M=K, and hence *D* is complex Gaussian distributed with conditional mean

(32) $$\begin{array}{c} m_{D}=\left(\right|e_{x}{|}^{2}+|e_{y}{|}^{2})\sum_{i=0}^{I-1}\;\sum_{k=0}^{K\;-\;2}\;|\tilde{s}{\left[i\right]|}^{2}{\left|p_{i}^{k}\left(u\right)\right|}^{2}e^{j(u-\frac{2\pi i}{L})} \end{array}$$

and conditional variance

(33) $$\begin{array}{c} \sigma_{D}^{2}=\sum_{i=0}^{I-1}\sum_{k=0}^{K-2}\left(\sigma_{{\tilde{N}}_{x}}^{2}+\sigma_{{\tilde{N}}_{y}}^{2}\right)=\frac{2(|e_{x}{|}^{2}+|e_{y}{|}^{2})\sigma_{n}^{2}}{K}\sum_{i=0}^{I-1}\sum_{k=0}^{K-2}|\tilde{s}\left[i\right]{|}^{2}{\left|p_{i}^{k}\left(u\right)\right|}^{2}. \end{array}$$

As a result, the conditional SNR of *D* given c, is given by

(34) $$\begin{array}{c} \gamma_{c}=\frac{|m_{D}{|}^{2}}{\sigma_{D}^{2}}=\frac{K(|e_{x}{|}^{2}+|e_{y}{|}^{2})}{2\sigma_{n}^{2}}\sum_{i=0}^{I-1}\sum_{k=0}^{K-2}|\tilde{s}{\left[i\right]|}^{2}{\left|p_{i}^{k}\left(u\right)\right|}^{2}. \end{array}$$

Taking the expectation of $\gamma_{c}$ over c and assuming d=λ/2, we have the average SNR of *D*, $\bar{\gamma }$, as

(35) $$\begin{array}{cc} \bar{\gamma }= & \frac{K\gamma_{s}}{2}\sum_{i=0}^{I-1}\;\sum_{k=0}^{K\;-\;2}\;E_{u,\gamma }\left\{|p_{i}^{k}{\left(u\right)|}^{2}\left(\sin^{2}\;\gamma \left(1\;-\;u^{2}/\pi^{2}\right)\;+\;\cos^{2}\;\gamma \right)\right\}\\ = & \frac{K\gamma_{s}}{2}\sum_{i=0}^{I-1}\sum_{k=0}^{K-2}E_{u}\left\{|p_{i}^{k}{\left(u\right)|}^{2}\right\}-\frac{K\gamma_{s}}{2\pi^{2}}\sum_{i=0}^{I-1}\sum_{k=0}^{K-2}E_{\gamma }\left\{\sin^{2}\gamma \right\}\cdot E_{u}\left\{|up_{i}^{k}{\left(u\right)|}^{2}\right\}\\ \overset{\left(a\right)}{=} & \frac{NI\left(K-1\right)\gamma_{s}}{2}-\frac{K\left(K-1\right)\gamma_{s}}{8\pi^{3}}\cdot \sum_{i=0}^{I-1}\int_{-\pi }^{\pi }{\left|\frac{u\sin\left(\frac{Nu}{2}-\frac{N\pi i}{L}\right)}{\sin\left(\frac{Ku}{2}-\frac{K\pi i}{L}\right)}\right|}^{2}du, \end{array}$$

where $\gamma_{s}=E\left\{\frac{|\tilde{s}{\left[i\right]|}^{2}}{\sigma_{n}^{2}}\right\}$, and γ is assumed to be uniformly distributed within (0,π/2), denoted by γ∼U(0,π/2). (*a*) holds because

(36) $$\begin{array}{c} E_{u}\left\{\right|p_{i}^{k}\left(u\right){|}^{2}\}=\frac{1}{2\pi }\int_{-\pi }^{\pi }{\left|\frac{\sin\left(\frac{Nu}{2}-\frac{N\pi i}{L}\right)}{\sin\left(\frac{Ku}{2}-\frac{K\pi i}{L}\right)}\right|}^{2}du=N/K \end{array}$$

assuming u∼U(−π,π).

Denote the probability density function (pdf) of $\hat{u}$ as $f_{\hat{u}}\left(\hat{u}\right)$. Assuming that $|\tilde{s}\left[i\right]|$ is Rayleigh distributed, we have $f_{\hat{u}}\left(\hat{u}\right)=f_{1}\left(\hat{u},\bar{\gamma }\right)$. At high SNRs, $f_{1}\left(\hat{u},\bar{\gamma }\right)$ is approximated as [13]

(37) $$\begin{array}{c} f_{1}\left(\hat{u},\bar{\gamma }\right)\approx \frac{\sqrt{\bar{\gamma }\pi^{2}+1}}{2\pi {\left(\bar{\gamma }{\hat{u}}^{2}+1\right)}^{3/2}},\;-\pi \leq \hat{u}<\pi . \end{array}$$

$f_{1}\left(\hat{u},\bar{\gamma }\right)$ is the true pdf of $\hat{u}$ if $|p_{i}^{k}\left(u\right)|$ reaches the maximum value *Q*. However, there will be an SNR reduction since $|p_{i}^{k}\left(u\right)|\leq Q$, ∀k,i,u. Furthermore, our derivation is based on the assumption of $\hat{Nu}=Nu$. Therefore, the actual MSE, $\sigma_{\hat{u}}^{2}$, will always be higher than that calculated using $f_{1}\left(\hat{u},\bar{\gamma }\right)$, i.e.,

(38) $$\begin{array}{cc} \sigma_{\hat{u}}^{2} & \geq \int_{-\pi }^{\pi }{\hat{u}}^{2}f_{1}\left(\hat{u},\bar{\gamma }\right)d\hat{u}=\frac{\sqrt{\bar{\gamma }\pi^{2}+1}}{\pi {\bar{\gamma }}^{3/2}}\sinh^{-1}\left(\sqrt{\bar{\gamma }}\pi \right)-\frac{1}{\bar{\gamma }}=\mathrm{MSELB}. \end{array}$$

As *I* and *K* increase, the MSELB is asymptotically tight. Similarly, when the signal reception is performed by a single-polarized hybrid array, the average SNRs of *D* using *x*- or *y*-axis dipoles only are given by ${\bar{\gamma }}_{x}=\bar{\gamma }-{\bar{\gamma }}_{y}$ and ${\bar{\gamma }}_{y}=\frac{NI\left(K-1\right)\gamma_{s}}{4}$, respectively. Substituting them into (38), it can be verified that the MSELB in [16] is a special case of our results.

### 5.2. Analysis of Polarization Estimation Performance

Since the polarization state information is included in $e_{x}e_{y}^{*}$ and $|e_{x}{|}^{2}+{\left|e_{y}\right|}^{2}$ of (22), they are considered as the wanted components of the numerator and denominator in (24), respectively, which are subject to scaling and additive noise.

The average SNR of $\sum_{i=0}^{I^{{}^{\prime }}-1}\sum_{m=0}^{M-1}\left(p_{x}^{m}\left[i\right]+p_{y}^{m}\left[i\right]\right)-2MI^{{}^{\prime }}\sigma_{n}^{2}$ for the estimation of $|e_{x}{|}^{2}+{\left|e_{y}\right|}^{2}$ in (24) is given by

(39) $$\begin{array}{cc} \; & E\left\{\frac{{\left|\left(\right|e_{x}{|}^{2}+|e_{y}{|}^{2})\sum_{i=0}^{I^{{}^{\prime }}-1}\sum_{m=0}^{M-1}|\tilde{s}{\left[i\right]|}^{2}{\left|P_{s}\left(u\right)\right|}^{2}\right|}^{2}}{2(|e_{x}{|}^{2}+|e_{y}{|}^{2})\sum_{i=0}^{I^{{}^{\prime }}-1}\sum_{m=0}^{M-1}|\tilde{s}{\left[i\right]|}^{2}{\left|P_{s}\left(u\right)\right|}^{2}\sigma_{n}^{2}}\right\}\\ \; & =\frac{|e_{x}{|}^{2}+{\left|e_{y}\right|}^{2}}{2}MI^{{}^{\prime }}\gamma_{s}E\left\{|P_{s}{\left(u\right)|}^{2}\right\}, \end{array}$$

while the average SNR of $\sum_{i=0}^{I^{{}^{\prime }}-1}\sum_{m=0}^{M-1}p_{x}^{m}\left[i\right]-MI^{{}^{\prime }}\sigma_{n}^{2}$ that uses *x*-axis dipole only to calculate the power as shown in [8], is given by

(40) $$\begin{array}{cc} \; & E\left\{\frac{{\left||e_{x}{|}^{2}\sum_{i=0}^{I^{{}^{\prime }}-1}\sum_{m=0}^{M-1}|\tilde{s}{\left[i\right]|}^{2}{\left|P_{s}\left(u\right)\right|}^{2}\right|}^{2}}{2|e_{x}{|}^{2}\sum_{i=0}^{I^{{}^{\prime }}-1}\sum_{m=0}^{M-1}|\tilde{s}{\left[i\right]|}^{2}{\left|P_{s}\left(u\right)\right|}^{2}\sigma_{n}^{2}}\right\}\\ \; & =\frac{|e_{x}{|}^{2}}{2}MI^{{}^{\prime }}\gamma_{s}E\left\{|P_{s}{\left(u\right)|}^{2}\right\}. \end{array}$$

It can be seen from (39) and (40) that our proposed approach has higher average SNR for the polarization parameter estimation than that using one axis dipole for power calculation, thus improving the estimation accuracy.

On the other hand, when the calculations of cross-correlation and power in (24) are performed before digital beamforming, the average SNR of $\sum_{i=0}^{I^{{}^{\prime }}-1}\sum_{m=0}^{M-1}q_{xy}^{m}\left[i\right]$ in (24) is given by

(41) $$\begin{array}{cc} \; & E\left\{\frac{{\left|e_{x}e_{y}^{*}\sum_{i=0}^{I^{{}^{\prime }}-1}\sum_{m=0}^{M-1}{\left|s\left[i\right]\right|}^{2}{\left|P_{s}\left(u\right)\right|}^{2}\right|}^{2}}{\left(\right|e_{x}{|}^{2}+|e_{y}{|}^{2})\sum_{i=0}^{I^{{}^{\prime }}-1}\sum_{m=0}^{M-1}{\left|s\left[i\right]\right|}^{2}{\left|P_{s}\left(u\right)\right|}^{2}\sigma_{n}^{2}}\right\}\\ \; & =\frac{|e_{x}{|}^{2}{\left|e_{y}\right|}^{2}}{|e_{x}{|}^{2}+{\left|e_{y}\right|}^{2}}MI^{{}^{\prime }}\gamma_{s}E\left\{|P_{s}{\left(u\right)|}^{2}\right\}. \end{array}$$

When the calculations of cross-correlation and power are performed after digital beamforming as shown in [8], i.e., the outputs of the digital beamformers of *x*- and *y*-axis dipoles are used for calculation, the average SNRs of the cross-correlation and power are given by

(42) $$\begin{array}{cc} \; & E\left\{\frac{{\left|e_{x}e_{y}^{*}\sum_{i=0}^{I^{{}^{\prime }}-1}{\left|s\left[i\right]\right|}^{2}|P_{s}{\left(u\right)|}^{2}{\left|P_{c}\left(\hat{u}\right)\right|}^{2}\right|}^{2}}{\left(\right|e_{x}{|}^{2}+|e_{y}{|}^{2})\sum_{i=0}^{I^{{}^{\prime }}-1}{\left|s\left[i\right]\right|}^{2}|P_{s}{\left(u\right)|}^{2}{\left|P_{c}\left(\hat{u}\right)\right|}^{2}M\sigma_{n}^{2}}\right\}\\ \; & =\frac{|e_{x}{|}^{2}{\left|e_{y}\right|}^{2}}{|e_{x}{|}^{2}+{\left|e_{y}\right|}^{2}}\cdot \frac{I^{{}^{\prime }}}{M}\gamma_{s}E\left\{|P_{s}{\left(u\right)|}^{2}\right\}E\left\{|P_{c}\left(\hat{u}\right){|}^{2}\right\} \end{array}$$

and

(43) $$\begin{array}{cc} \; & E\left\{\frac{{\left|\left(\right|e_{x}{|}^{2}+|e_{y}{|}^{2})\sum_{i=0}^{I^{{}^{\prime }}-1}|\tilde{s}{\left[i\right]|}^{2}|P_{s}{\left(u\right)|}^{2}{\left|P_{c}\left(\hat{u}\right)\right|}^{2}\right|}^{2}}{2(|e_{x}{|}^{2}+|e_{y}{|}^{2})\sum_{i=0}^{I^{{}^{\prime }}-1}|\tilde{s}{\left[i\right]|}^{2}|P_{s}{\left(u\right)|}^{2}{\left|P_{c}\left(\hat{u}\right)\right|}^{2}M\sigma_{n}^{2}}\right\}\\ \; & =\frac{|e_{x}{|}^{2}+{\left|e_{y}\right|}^{2}}{2}\cdot \frac{I^{{}^{\prime }}}{M}\gamma_{s}E\left\{|P_{s}{\left(u\right)|}^{2}\right\}E\left\{|P_{c}\left(\hat{u}\right){|}^{2}\right\}, \end{array}$$

respectively, where $P_{c}\left(\hat{u}\right)=\sum_{m=0}^{M-1}e^{jm\hat{u}}=\frac{\sin M\hat{u}/2}{\sin\hat{u}/2}$. By comparing (39) and (41) to (42) and (43), we can see that our proposed approach that calculates the cross-correlation and power before digital beamforming outperforms that after digital beamforming in terms of the average SNR for polarization estimation since $|P_{c}\left(\hat{u}\right){|}^{2}\leq M^{2}$.

## 6. Numerical and Simulation Results

In this section, we present the numerical and simulation results of AoA and polarization parameter estimation to evaluate the proposed approaches using hybrid dual-polarized arrays. The state of the art [16] using hybrid single-polarized arrays is also simulated for comparison. Denote the average SNR per EMVS as $\gamma_{a}$, which is given by $\gamma_{a}=N\gamma_{s}$. The reference signal, $\tilde{s}\left(t\right)$, is generated following complex Gaussian distributions. Considering the reception of polarized signals following u∼U(−π,π), γ∼U(0,π/2) and η∼U(−π,π), simulation results are obtained by averaging over 50,000 independent trials.

**Figure 2:**
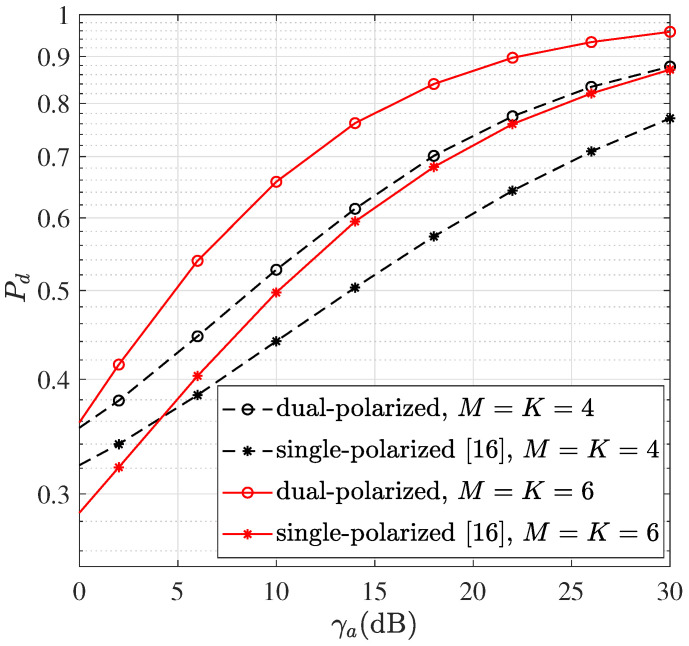
Detection probability of $m^{\prime }$, $P_{d}$ versus $\gamma_{a}$, where N=12 and I=8.

Figure 2 compares the detection probability of $m^{\prime }$, $P_{d}$, versus $\gamma_{a}$ using dual-polarized and single-polarized (*x*-axis dipoles) arrays, where the number of EMVSs in each subarray, *N*, is fixed to be 12 and the number of reference signals, *I*, is set to be 8. We define the probability of finding the index $m^{\prime }$ of differential signals with the largest amplitude as $P_{d}$. As shown in the figure, the dual-polarized array with EMVS is superior to the single-polarized array in [16] that only uses one axis dipoles in terms of $P_{d}$. It indicates that the use of dual-polarized arrays has a better calibration capability, and thus improving the accuracy of $\hat{Nu}$. Moreover, using more subarrays leads to higher detection probability with the increase of $\gamma_{a}$. Given the number of antennas in a subarray, *N*, as the number of subarrays, *M*, increases, more beams will achieve higher SNR for cross correlations and thus more accurate $m^{\prime }$. Because the number of *N* determines the width of each beam, the boundary of *M* depends on *N*. The increasing *N* generally results in a larger *M*.

**Figure 3:**
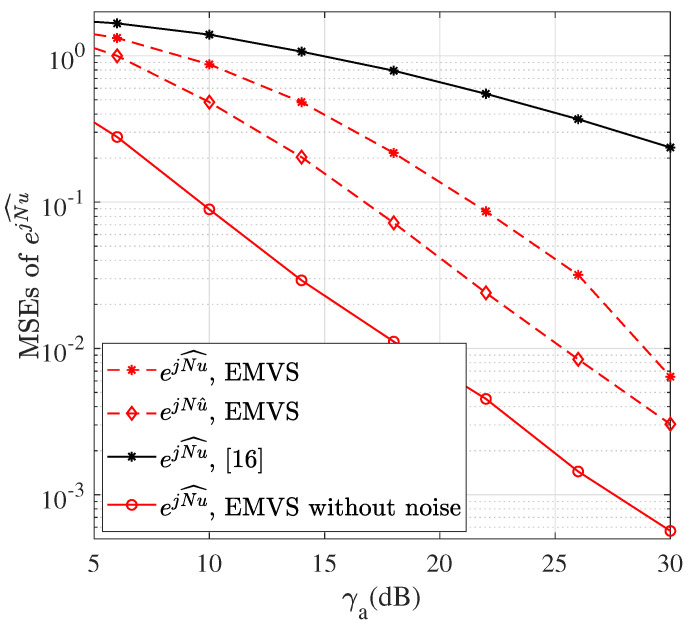
The MSEs of $e^{j\hat{Nu}}$ versus $\gamma_{a}$.

The MSEs of the estimates are shown as a function of $\gamma_{a}$ in Figure 3, where M=K=4 and N=I=8. It can be seen that the MSEs of $e^{j\hat{Nu}}$ (refer to Step 10 in Algorithm 1) using dual-polarized arrays are lower than those using single-polarized arrays in [16], which is in line with the results in Figure 2. The MSE performance is also greatly enhanced from $e^{j\hat{Nu}}$ to $e^{jN\hat{u}}$, e.g., a 4 dB improvement at the MSE of 0.1. The MSEs of $e^{j\hat{Nu}}$ without noise is also plotted as a comparison.

**Figure 4:**
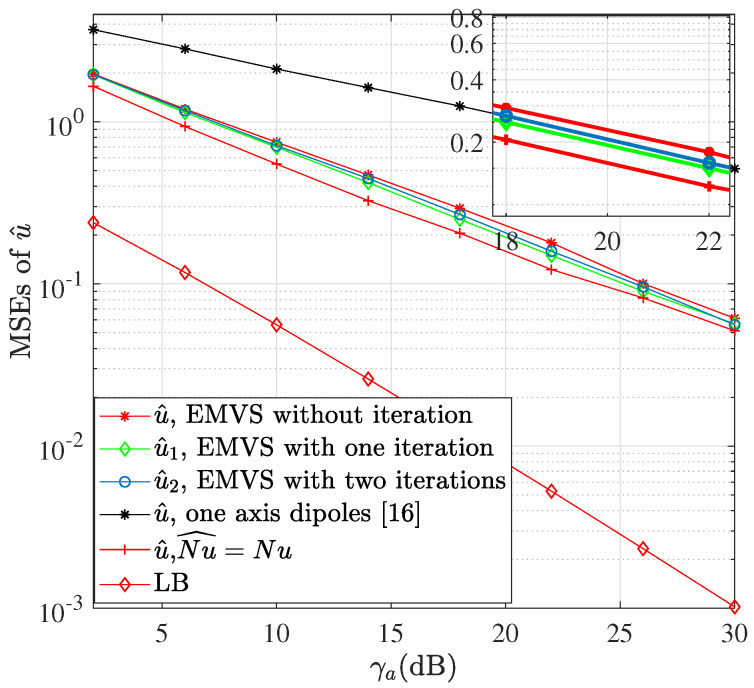
The MSEs of $\hat{u}$ versus $\gamma_{a}$.

Figure 4 shows the MSEs of $\hat{u}$ versus $\gamma_{a}$. It is seen that the MSEs of $\hat{u}$ (refer to Step 22 in Algorithm 1) with dual-polarized arrays are lower than those of single-polarized arrays in [16]. The MSE performance of ${\hat{u}}_{1}$ is improved after one iteration due to the enhanced $\hat{Nu}$ (refer to the updated estimate at step 23), while the MSE performance of ${\hat{u}}_{2}$ after two iterations has no further improvement. The MSEs of $\hat{u}$ are displayed for comparison assuming $\hat{Nu}=Nu$, which means that the signals from all subarrays’ outputs are perfectly aligned. The MSELBs exhibit far better performance due to assuming $|p_{i}^{k}\left(u\right)|=Q$, ∀k,i. However, only few of them can be close to *Q* and even some ones are far less than *Q* in terms of the phase shift designs in this paper. Therefore, there is a certain gap between the MSE performance of the proposed AoA estimation approach and the MSELBs.

**Figure 5:**
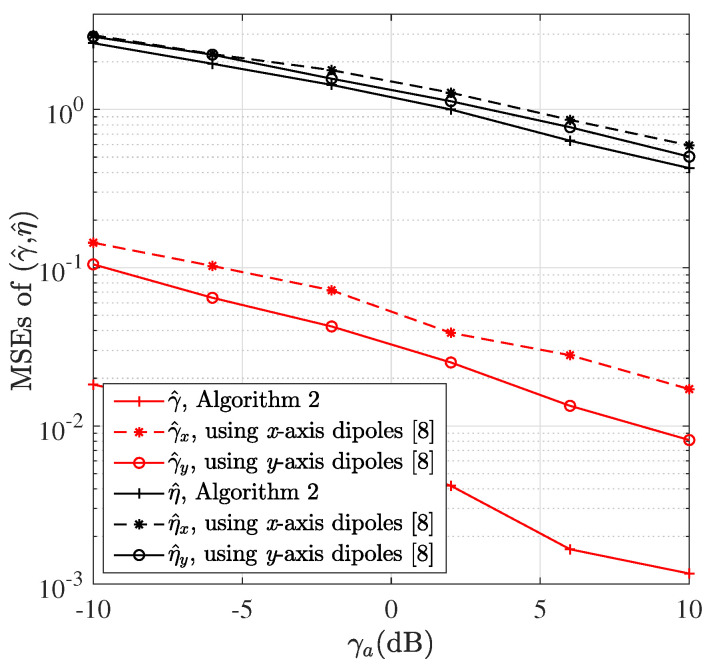
The MSEs of ($\hat{\gamma }$,$\hat{\eta }$) versus $\gamma_{a}$.

Figure 5 shows the MSEs of polarization parameters versus $\gamma_{a}$, where N=8 and M=6. It can be seen that the MSEs of $\hat{\gamma }$ and $\hat{\eta }$ using Algorithm 2 for power calculation are lower than those using only one axis dipoles for power calculation in [8], which is consistent with the performance analysis in Section 5.2.

**Figure 6:**
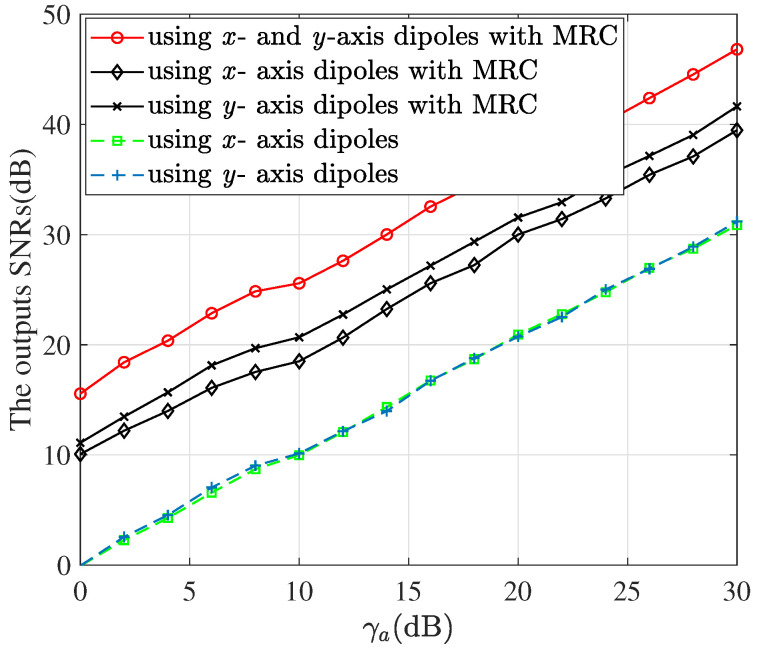
The output SNRs versus the $\gamma_{a}$, where N=8 and M=6.

Figure 6 shows the SNRs of the combined signal defined in (4) with MRC, and of the signals only using *x*- or *y*- dipoles without MRC. The circle marker denotes the output SNRs with MRC using the total received power of signals on *x*- and *y*-axis dipoles for the cross-correlation-to-power ratio calculation, while the diamond or cross marker denotes those with MRC using *x*- or *y*-axis dipole for ratio calculation. It is seen that the former achieves higher output SNRs than the latters due to the improved accuracy of polarization parameter estimation, i.e., more accurate MRC coefficients. The hybrid dual-polarized array with MRC produces far higher output SNRs than the single-polarized array with one axis dipoles without MRC. Note that the simulated output SNR using *x*- and *y*-axis dipoles with MRC at $\gamma_{a}=10$ dB is given by 26.4 dB. It is close to the SNR that is achieved by the arrays perfectly aligned with the real AoA and polarization state, given by 10 + 10 lg(6 × 8) = 26.8 dB.

## 7. Conclusions

In this paper, we have developed enhanced AoA and polarization parameter estimation approaches for localized mmWave hybrid dual-polarized arrays. Employing the polarization diversity, dual-polarized antennas can effectively enhance the calibration capability of the signs of differential signals, and thus the SNR for AoA estimation. Based on the enhanced AoA estimate, the proposed cross-correlation-and-power ratio based approach exploiting the total power of EMVS can greatly improve the accuracy of polarization estimation. Furthermore, we have provided the closed-form MSELBs for the enhanced AoA estimation and analytical performance evaluation of the enhanced polarization estimation approach. Simulation results show that the output SNRs of the MRC signals can be effectively improved with the enhanced AoA and polarization parameter estimation. In future work, the proposed algorithms have potentials to be extended to the frequency domain for estimation accuracy improvement.

## Data Availability

Not applicable.
